# Supplementary material for: Content-rich biological network constructed by mining PubMed abstracts
Source: BMC Bioinformatics. 2004 Oct 8;5:147. doi: 10.1186/1471-2105-5-147 (PMC528731; doi:10.1186/1471-2105-5-147)
Supplement: Additional File 2 — The original results of the above study (non-essential files are deleted to keep the file size under the limit set by BMC bioinformatics). [file 1471-2105-5-147-S2.bz2 › chilibotAdditionalFile2/dip10/9ID9009191E30/html/ACTN2_GRIN1.html]

 


 **ACTN2** and **GRIN1** 
  
Found 10 abstracts in PubMed, retrieved 10.  
 

 What does Google say? 
 PDF only 
| .edu only 

---

**Interactive relationship** (e.g. stimulation, inhibition, etc)

**Inhibitory relationship**- Furthermore, this inactivating effect of calmodulin can be prevented by coexpressing a region of the cytoskeletal protein alpha actinin2  [ **ACTN2** ]  known to interact with the CO region of NR1  [ **GRIN1** ] .  Ref: 9728925 Neuron, 1998
- A similar reduction in the effects of ethanol on wild type NR1  [ **GRIN1** ]  2A but not NR1  [ **GRIN1** ]  2B or NR1  [ **GRIN1** ]  2C receptors was observed after co expression of full length or truncated human skeletal muscle alpha actinin 2  [ **ACTN2** ]  proteins that produce a functional knockout of the C0 domain.  Ref: 10809744 J Biol Chem, 2000
**Neutral relationship**- Alpha actinin alpha actinin 2  [ **ACTN2** ]  is a protein which links the NR1  [ **GRIN1** ]  and NR2B subunits of N methyl D aspartate NMDA glutamate receptors to the actin cytoskeleton.  Ref: 10925145 Brain Res Mol Brain Res, 2000
- Conversely, alpha actinin 2  [ **ACTN2** ]  antibody immunoprecipitated only modest fractions of striatal NR1  [ **GRIN1** ] , NR2A and NR2B subunits.  Ref: 10925145 Brain Res Mol Brain Res, 2000
- Additionally, the limited expression of alpha actinin 2  [ **ACTN2** ]  could have important functional implications in N methyl D aspartate receptor  [ **GRIN1** ]  localization and modulation.  Ref: 11246149 Neuroscience, 2001
- alpha Actinin 2  [ **ACTN2** ]  binds by its central rod domain to the cytoplasmic tail of both NR1  [ **GRIN1** ]  and NR2B subunits of the NMDA receptor, and can be immunoprecipitated with NMDA receptors and PSD 95 from rat brain.  Ref: 9009191 Nature, 1997
- A subunit specific anti NR1  [ **GRIN1** ]  antibody co precipitated major fractions of NR2A and NR2B subunits, but only a minor fraction of striatal alpha actinin 2  [ **ACTN2** ] .  Ref: 10925145 Brain Res Mol Brain Res, 2000

**Non-interactive relationship** (e.g. studied together, co-existance, homology, etc.)

- Recently the actin binding protein alpha actinin 2  [ **ACTN2** ]  was shown to bind directly to the NMDA receptor subunits NR1  [ **GRIN1** ]  and NR2B, suggesting that alpha actinin 2  [ **ACTN2** ]  may function to attach NMDA receptors to the actin cytoskeleton.  Ref: 9454847 J Neurosci, 1998
- By immunogold electron microscopy, alpha actinin 2  [ **ACTN2** ]  is concentrated over the postsynaptic density PSD of numerous asymmetric synapses where it colocalizes with NR1  [ **GRIN1** ]  immunoreactivity.  Ref: 9454847 J Neurosci, 1998
- Differential immunoreactivity for alpha actinin 2  [ **ACTN2** ] , an N methyl D aspartate receptor  [ **GRIN1** ]  actin binding protein, in hippocampal interneurons.  Ref: 11246149 Neuroscience, 2001
- Reduced ethanol inhibition of N methyl D aspartate receptors by deletion of the NR1  [ **GRIN1** ]  C0 domain or overexpression of alpha actinin 2  [ **ACTN2** ]  proteins.  Ref: 10809744 J Biol Chem, 2000
- This study examined the immunoreactivity of alpha actinin 2  [ **ACTN2** ] , an actin binding N methyl D aspartate receptor  [ **GRIN1** ]  linking protein, in the rat hippocampal formation using double labelling immunofluorescence.  Ref: 11246149 Neuroscience, 2001
- Like Ca calmodulin, autophosphorylated CaMKII competes with alpha actinin 2  [ **ACTN2** ]  for binding to NR1  [ **GRIN1** ] .  Ref: 12379661 J Biol Chem, 2002
